# The effect of spiritual healing on *in vitro* tumour cell proliferation and viability – an experimental study

**DOI:** 10.1038/sj.bjc.6602749

**Published:** 2005-08-23

**Authors:** R Zachariae, L Højgaard, C Zachariae, M Væth, B Bang, L Skov

**Affiliations:** 1 Department of Oncology, Aarhus University Hospital, 8000 Århus C, Denmark; 2 Danish Healing Research Center, Stationsvej 16, 3210 Vejby, Denmark; 3 Department of Dermatology, Copenhagen University Hospital, 2820 Gentofte, Denmark; 4 Department of Biostatistics, Aarhus University, 8000 Århus C, Denmark

**Keywords:** healing, alternative medicine, MCF-7, HB-94

## Abstract

Alternative treatments such as spiritual healing and prayer are increasingly popular, especially among patients with life-threatening diseases such as cancer. According to theories of spiritual healing, this intervention is thought to influence living cells and organisms independently of the recipient's conscious awareness of the healer's intention. The aim of this study was to test the hypothesis that spiritual healing will reduce proliferation and viability of two cancer cell lines *in vitro*. Three controlled experiments were conducted with three different healers and randomised allocation of cells to five different doses of healing or control. Researchers conducting the assays and statistical analyses were blinded to the experimental conditions. Main outcome measures were MTT viability, ^3^H-thymidine incorporation and counts of an adherent human breast cancer cell line (MCF-7), and a nonadherent mouse B-lymphoid cell line (HB-94). Analyses of variance (ANOVAs) revealed no significant main or dose-related effects of spiritual healing compared to controls for either of the two cell lines or any of the assays (*P*-values between 0.09 and 0.96). When comparing healing and control across all three experimental days, doses, assays, and cells, 34 (51.6%) of 66 independent comparisons showed differences in the hypothesised direction (*P*=0.90). The average effect size across cell lines, days, assays, and doses approached zero (Cohen's *d*=−0.01). The results do not support previous reports of beneficial effects of spiritual healing on malignant cell growth *in vitro*. Reported beneficial effects of spiritual healing on the well-being of cancer patients seem more likely to be mediated by psychosocial and psychophysiological effects of the healer–patient relationship.

Complementary and alternative medicine (CAM) (National Center for Alternative and Complementary Medicine, 2004) is increasingly popular, especially among patients with chronic and life-threatening diseases such as cancer (Lerner and Kennedy, 1992; Downer *et al*, 1994; Boon *et al*, 2000). Results from a 1999 national health interview survey of the use of CAM showed that spiritual healing or prayer was the most frequently used complementary or alternative treatment (13.7%) by adults in the US (Ni *et al*, 2002). Spiritual healing has been defined as a systematic, purposeful intervention by one or more persons aiming to help another living being (person, animal, plant, cell or other living system) by means of focused intention to improve their condition (Benor, 2002). Spiritual healing includes many categories, for example, ‘therapeutic touch’ and ‘intercessory prayer’, and the healing may be attributed to God, spirits, universal forces or energies, biological healing energies residing in the healer, or self-healing powers or energies thought to reside latent in the healed organism.

A review suggests that spiritual healing may have beneficial effects on various human psychological and physical problems (Benor, 2002). The results, however, are mixed, and the methodological quality often questionable. A major problem in interpreting the results of human healing research lies in controlling for the influence of factors other than the hypothesised specific effect of spiritual healing, defined as influencing at a ‘distance’, through ‘nonlocal consciousness’ independently of the recipients awareness of the healers intention. Such other factors may include ‘nonspecific’ effects (Frank, 1973) of beliefs, expectation, and other psychological aspects of the healer–patient relationship. Such confounders are not an issue in experiments carried out *in vitro*, and results from experiments studying the effects of spiritual healing on bacteria (Nash, 1982), yeasts (Haraldsson and Thorsteinsson, 1973), microorganisms (Pleass and Dey, 2005), or human cells in the laboratory (Braud, 1990) have been published. While the majority of these reports show positive results, replications are rare.

A search of the literature revealed three studies of effects of healing on malignant cells *in vitro*. Chen and He (2001) reported significantly reduced PPT-I expression in breast cancer cells following Qigong, a Chinese form of healing. Shah *et al* (1999) reported moderate effects of healing in the expected direction, that is, reduced cell counts, for four different tumour cell lines. Finally, in an early study, Snel (1980) investigated the effect of healing on growth of mouse leukaemia cells *in vitro*. One of three experiments yielded results in the expected direction. The few available results do not allow for a clear conclusion. Although other studies have been conducted over the last 15 years, the results are only available as abstracts from conference proceedings or unpublished preliminary reports.

The highly controversial hypothesised effects of distant or spiritual healing contradict our ordinary sense of reality, are in conflict with the generally agreed upon laws of science, and only the results of independent, well-controlled experimental studies can clarify the issue. The aim of our study was therefore to investigate the possible inhibiting effects of spiritual healing on cell counts, viability, and proliferation of two cancer cell lines with different characteristics: human and animal, adherent and nonadherent. A human adherent breast cancer cell line was chosen, since breast cancer represents one of the leading causes of cancer-related death, and since positive results had previously been reported for breast cancer cell lines (Shah *et al*, 1999; Chen and He, 2001). In addition, a nonadherent cell line of mouse origin was used. Together with cell counting, as investigated in most previous studies, we included assays measuring two additional aspects of cell growth.

## METHODS

### Cell lines and assays

#### Cell lines

Two cell lines were used: (1) MCF-7, an adherent human breast cancer cell line (Brooks *et al*, 1973) and (2) HB-94, a nonadherent mouse B-lymphoid cell line (Brodsky and Parham, 1982) (ATCC, Rockville, MD, USA). *Cell viability*: The 3-(4,5-dimethylthiazole-2-yl)-2,5-diphenyltetrazolium bromide (MTT) reduction assay (MTT-assay) was used for quantification of viable cells (Mosmann, 1983). *Cell proliferation* was measured using a standard ^3^H-thymidine incorporation assay. *Cell counts* were performed independently in a haemocytometer by two researchers (BB and LS). The cells were counted and viability and proliferation assays were conducted the following day, 24 h after beginning the experiment. In the previous studies, incubation periods were 16 h (four breast cancer cell lines) (Chen and He, 2001), 24 h (four different cell lines, including the MCF-7 breast cancer cell line) (Shah *et al*, 1999), and 96 h (mouse leukaemia cells) (Snel, 1980). In the latter study, only one of four experiments showed changes in the expected direction.

### Procedure

A total of 12 plates were used in each of three experiments under identical conditions on three separate days. In all, 10 plates were randomised to an experimental (healing) or a control group, and two plates remained in the incubator during the experiment as additional control. Experimental and control plates were removed from the incubator and placed in a flow bench for the same number of minutes with the same time interval in counter-balanced order. All 10 plates were placed in a flow bench for 10 min followed by 50 min in the incubator, eight plates for an additional 10 min, six plates for 3 × 10 min, four plates for 4 × 10 min, and two plates for a total of 5 × 10 min, yielding a design with increasing doses of exposure (10, 20, 30, 40, and 50 min) to healing or control over a total of 4 h and 10 min for each condition. A total of 504 wells were analysed, making it possible to compare cells in 2 × 45 wells across all experimental days and doses for each cell line and assay. Setting aside a total of 84 wells for additional control, a total of 2 × 15 wells were available for cell counting for each cell line.

### Healing

Healing was performed by three healers with 6–18 years of experience, all reporting themselves as successful in their clinical work. None had previously worked with biological material. The healers used their own individual method to attempt to inhibit viability and growth of the cancer cells. Two thus held their hands 20–30 cm over the plates, but none of the healers touched the plates physically at any time. The sessions were video recorded, and after the sessions, the healers were interviewed concerning their experiences during the experiment. The healers were not present during control sessions.

### Blinding

The researchers involved in the analysis of the cell cultures were blinded with respect to experimental condition and dose. The statistician (MV) was blinded to the purpose of the study and the experimental conditions.

### Power considerations

Data from the three previous experiments involving malignant cell cultures showed significant differences between healing and control corresponding to effect sizes (Cohen's *d* (Cohen, 1988)) ranging from −0.5 to +2.0 (average: +0.6) (Snel, 1980; Shah *et al*, 1999; Chen and He, 2001). With the number of plates possible in our study, simple comparisons across all three experimental days and all five doses allowed us to detect a difference corresponding to an effect size of 0.6 with a statistical power of 80% (*α*: 5%) for each assay and cell line. Setting aside 84 wells for additional control cells to be kept in the incubator, a total of 2 × 15 wells remained available for cell counting for both cell lines, making it possible to detect a difference corresponding to an effect size of 1.06 with a statistical power of 80% (*α*: 5%).

### Statistical methods

Analysis of variance (ANOVA) methods were used to analyse the experimental data. Each cell line was analysed separately for each of the three assays. Data from the unmanipulated control plates were not included in these analyses. Non-normally distributed data were log transformed prior to analysis. The ANOVA used to analyse MTT viability and ^3^H-thymidine incorporation was based on the average of measurements from the three wells and included main effects of condition (healing and control) and dose (10–50 min), and an interaction between these factors. The effect of healing was assessed by testing the hypotheses of no interaction between dose and condition and no main effect of condition. The experimental design for the cell count experiments was different, and the analysis here was based on the difference between counts for the two conditions. The effect of healing was assessed by testing the hypotheses of no main effect of dose. The main effect of healing was evaluated by computing a confidence interval for the overall mean of the differences. In a supplementary analysis, dose was included as a covariate to assess a linear trend with dose. To investigate differences between healers these analyses were supplemented by ANOVAs, which included day (=healer) and interactions with day as systematic effects. The statistical package GenStat Release 7.1 (VSN International Ltd, UK) was used for all computations. To allow for comparison with previous results, effect sizes (Cohen's *d*) were calculated for the difference between healing and control for each assay, cell line, and dose, as well as for the pooled differences across doses for counts for both cell lines.

(*Additional information on assays, procedure and statistical analyses is available on request to the corresponding author*.)

## RESULTS

**Figure 1:**
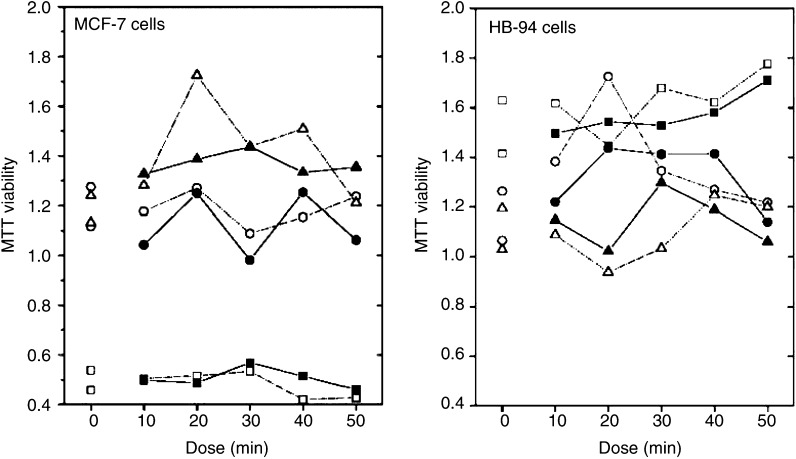
Average MTT-viability measurements (counts per ml) by day, condition, and dose (10–50 min). Left panel shows results for MCF-7 cells, right panel shows results for HB-94 cells. Experimental units, which received healing, are shown with filled symbols, control units with open symbols. Symbols: Square for day 1, circle for day 2, and triangle for day 3. Dose ‘0’ represents additional controls remaining in the incubator.

The results of the ANOVA of the MTT viability measurements are summarised in Table 1. Figure 1 shows the average results for each day, dose, and condition. For the MCF-7 cells, the dependence on dose did not differ between the two experimental conditions (*P*=0.76), and the overall difference was not statistically significant (*P*=0.35). When dose was included as a covariate, the linear trend with dose did not differ between the two experimental conditions (*P*=0.41) and no overall trend with dose was found (*P*=0.53). The findings were very similar for the HB-94 cell line. Again, the dose dependence did not depend on experimental condition (*P*=0.73), the overall difference was not statistically significant (*P*=0.53), the trend with dose did not depend on dose (*P*=0.96), and the overall trend was not statistically different from zero (*P*=0.69). For both cell lines, we found a considerable variation between days/healers (see Figure 1), but a similar day-to-day variation was also seen for the unexposed control measurements.

**Figure 2:**
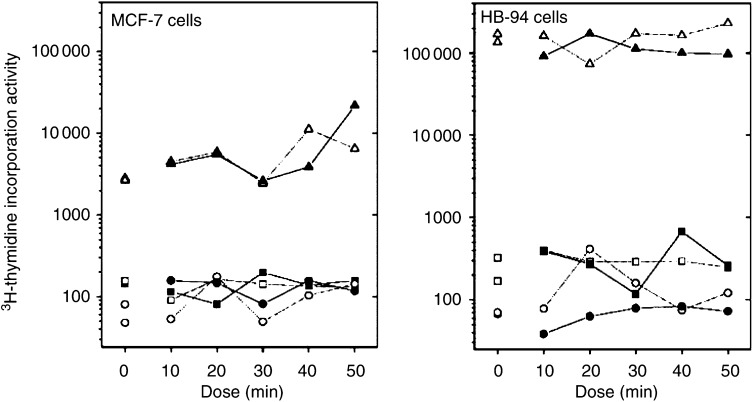
Average ^3^H-thymidine incorporation activity (counts per ml) by day, condition, and dose (10–50 min). Left panel shows results for MCF-7 cells, right panel shows results for HB-94 cells. Experimental units, which received healing, are shown with filled symbols, control units with open symbols. Symbols: square for day 1, circle for day 2, and triangle for day 3. Dose ‘0’ represents additional controls remaining in the incubator.

Table 2 gives the corresponding results for ^3^H-thymidine incorporation activity, and Figure 2 shows the average activity for each day, dose, and condition. For both cell lines, the dose dependence did not differ between the two experimental conditions (MCF-7: *P*=0.39, HB-94: *P*=0.74), and no statistically significant, overall difference was found (MCF-7: *P*=0.45, HB-94: *P*=0.09). For both cell lines, these conclusions were maintained when the dose dependence was described by a linear trend. As found for MTT viability, we found considerable variation between days/healers for the ^3^H-thymidine incorporation activity (Figure 2), and again this variation was also present for the unexposed control measurements.

**Figure 3:**
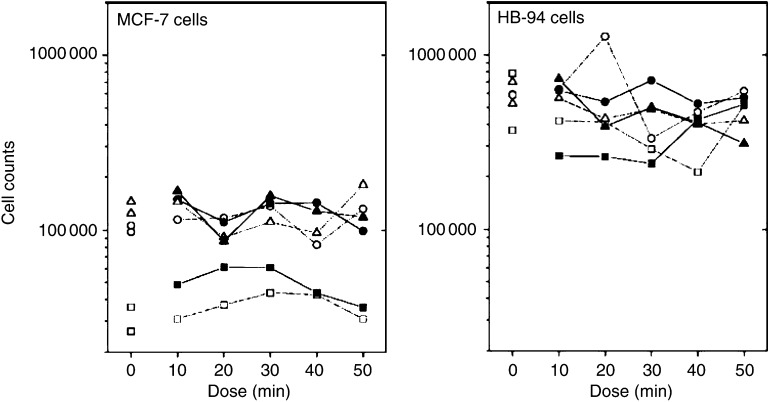
Average cell count per ml by day, condition, and dose (10–50 min). Left panel shows results for MCF-7 cells, right panel shows results for HB-94 cells. Experimental units, which received healing, are shown with filled symbols, control units with open symbols. Symbols: square for day 1, circle for day 2, and triangle for day 3. Dose ‘0’ represents additional controls remaining in the incubator.

Table 3 presents the results of the ANOVA of the cell counts and Figure 3 shows the average count for each day, dose, and condition. For both cell lines, the contribution to the total variation of the variation between doses within days was statistically significant. The dependence of the differences on dose was therefore evaluated relative to this random component. The analysis showed that the effect of dose was not statistically significant (MCF-7: *P*=0.19, HB-94: *P*=0.28). When dose was included as a covariate, no linear trend with dose was seen in the difference between the two experimental conditions (MCF-7 cells: *P*=0.13, HB-94 cells: *P*=0.45). For cell counts, the day-to-day variation was less striking, but also present in the unexposed control measurements.

Finally, standardised differences between healing and control, that is, effect sizes (Cohen's *d*) (Cohen, 1988), were calculated for each of 66 possible independent comparisons across days (healers), cell lines, assays, doses, and cell lines (*data not shown, but available on request to the corresponding author*). Of 66 independent comparisons, 34 (51.6%) yielded results in the expected direction, corresponding to a probability of 0.90 (two-tailed). The per cent differences in the expected direction for each of the 3 days were 50% (Day 1), 55% (Day 2), and 55% (Day 3), with the corresponding probabilities of 1.00, 0.83, and 0.83. The average effect size across all cell lines, days, assays, and doses approached zero (*d*=−0.01).

Two data points were missing and two extreme outliers were identified (*data not shown, details concerning raw data available on request*), representing a total data loss of less than 1%.

## DISCUSSION

This study did not reveal any significant main or dose-related effect of spiritual healing on proliferation and viability of either of two cancer cell lines. While there was considerable variability between days, there were no differences between days in the dose-dependent effects of healing and control. Across cell lines, experimental days, assays, and doses, an almost equal number of differences were in the hypothesised (51.6%) and the opposite of the hypothesised direction (48.4%). Our results differ from those of three previous experimental studies of healing and cancer cells *in vitro* (Snel, 1980; Shah *et al*, 1999; Chen and He, 2001).

### Strengths and weaknesses

A systematic review of published healing research (Crawford *et al*, 2003) indicates that many studies fail to fulfil important quality criteria, including (a) adequate control, (b) randomisation, (c) baseline comparability, (d) blinding, (e) acceptable loss of data, (f) clear description of intervention, (g) outcome measures, (h) adequate statistical analysis, and (i) reproducibility. In the present study, all the above criteria were met. In addition to control cells being handled similarly to the cells exposed to healing, that is, moving the cells from and back to the incubator, additional controls remained in the incubator. These additional controls (dose ‘0’) did not differ from cells in the intervention and control group. Special care was taken in blinding not only the researchers conducting the biological assays but also the statistician. Data loss was minimal, and reproducibility was met by conducting three separate experiments with three different healers under identical conditions, using two cell lines and three biological indicators of cell proliferation and viability. In contrast to previous studies (Snel, 1980; Shah *et al*, 1999; Chen and He, 2001), increasing doses of healing were included, enabling detection of a possible dose-dependent relationship. Still, the large number of wells yielded adequate statistical power to detect differences across doses similar to or smaller than those found in the previous studies.

One explanation for our negative findings could be that the doses used (10–50 min) were inadequate. They were, however, generally not smaller than those used in previous studies reporting significant effects of spiritual healing. For example, in one study with reported effects on malignant cell growth, the healing dose was reported as 20 min (Shah *et al*, 1999), and in a study reporting effects of healing on cytotoxic activity of natural killer cells doses were from 30 s to 5 min (Lee *et al*, 2001). The healers used the healing method of their own choice, and two of the healers chose to hold their hands approx. 20–30 cm over the plates, while the third healer did not. This could theoretically be a cause of concern. It has been proposed that healing effects could be due to electromagnetic fields (Seto *et al*, 1992) or infrared radiation (Chien *et al*, 1991) emitted from the hands of the healer. However, in spite of considerable day-to-day variation, we found no differences in the effects of healing across healers. Also, the additional controls remaining in the incubator showed similar variation, indicating that this was due to factors other than the intervention.

It is theoretically possible that the effects of healing could be transient or delayed, and that positive results would have been found if other incubation periods had been used. However, the incubation period used (24 h) was similar to the ones used in previous studies reporting effects of healing on malignant cells in culture (16–24 h). Moreover, if our negative results were a result of too extensive or insufficient timing, we should expect at least some indication of an effect in the expected direction. This was clearly not the case.

In addition to conventional methodological issues, other specific issues may be relevant for healing research (Schlitz *et al*, 2003). Since ‘healing intention’ is a key element in spiritual healing, a positive result may require that the healer has both the desire to heal and the belief and skill necessary to perform the healing. When interviewed after the experiment, the healers generally reported no concerns regarding their intention, desire, or skills. Finally, it could still be argued that the mere fact that an experiment was conducted could limit the ability of the healers. Informed of the negative result, one healer involved thus suggested that a ‘selfish intention to prove that healing works’ differs from the true altruistic ‘intention to heal’, and that only the latter should be expected to show results. Also, it should be noted that tumour cells differ in their characteristics, and it is possible that some cell types are more susceptible to healing than others.

## CONCLUSIONS

While agreeing that psychological aspects are a part of healing in the human setting, proponents of spiritual healing stress that effects are due to mechanisms beyond conventional pharmacological, physiological and psychological factors, and that healing can be performed ‘at a distance’ without conscious awareness on the part of the recipient, which can be any living being (person, animal, plant, cell, or other living system) (Benor, 2002). However, in the present tightly controlled study there was no evidence of such an effect for the cell lines investigated. The theory of spiritual healing is highly controversial and in conflict with the generally agreed upon laws of science. While this could explain why spiritual healing and other nonconventional methods are rarely investigated, the lack of scientific research will continue to leave the proposed mechanisms and effects of such interventions generally unchallenged.

## Figures and Tables

**Table 1:** MTT-viability assay

	**MCF-7 cells**				**HB-94 cells**			
**Source of variation**	**Mean square**	**df**	**F-test**	***P*-value**	**Mean square**	**df**	**F-test**	***P*-value**
Between days	2.196	2	197.01	<0.001	0.570	2	15.98	<0.001
								
*Day by dose stratum*								
Dose	0.020	4	1.83	0.217	0.004	4	0.11	0.976
Residual	0.011	8	1.14		0.036	8	3.01	
								
*Day by dose by condition stratum*								
Condition	0.009	1	0.97	0.349	0.005	1	0.43	0.526
Condition × dose	0.005	4	0.46	0.762	0.006	4	0.51	0.732
Residual	0.010	10			0.012	10		

Analysis of variance of average readings.

**Table 2:** ^3^H-thymidine incorporation assay

	**MCF-7 cells**				**HB-94 cells**			
**Source of variation**	**Mean square**	**df**	**F-test**	***P*-value**	**Mean square**	**df**	**F-test**	***P*-value**
Between days	9.185	2	192.86	<0.001	28.451	2	553.94	<0.001
								
*Day by dose stratum*								
Dose	0.090	4	1.9	0.204	0.010	4	0.2	0.934
Residual	0.048	8	1.53	0.260	0.051	8	0.98	0.502
								
*Day by dose by condition stratum*								
Condition	0.019	1	0.63	0.447	0.178	1	3.41	0.094
Condition × dose	0.036	4	1.15	0.390	0.026	4	0.5	0.736
Residual	0.031	10			0.052	10		

Analysis of variance of average log-transformed activity.

**Table 3:** Cell counts

	**MCF-7 cells**				**HB-94 cells**			
**Source of variation**	**Mean square**	**df**	**F-test**	***P*-value**	**Mean square**	**df**	**F-test**	***P*-value**
Between days	0.029	2	9.32	0.004	0.002	2	0.30	0.746
Between persons	0.013	1	8.96	0.011	0.019	1	2.30	0.155
Days by person stratum	0.001	2	0.48	0.630	0.008	2	1.04	0.384
								
*Day by dose stratum*								
Dose	0.046	4	2.01	0.186	0.101	4	1.56	0.275
Residual	0.023	8	7.43	0.001	0.065	8	7.91	0.001
								
Day by person by dose stratum	0.003	12			0.008	12		

Analysis of variance of differences between log-transformed counts.
